# The Design of a Multi-component Intervention to Promote Screening Mammography in an American Indian Community: The Native Women's Health Project

**DOI:** 10.3934/publichealth.2016.4.933

**Published:** 2016-11-18

**Authors:** Eleni L. Tolma, Kimberly Engelman, Julie A. Stoner, Cara Thomas, Stephanie Joseph, Ji Li, Cecily Blackwater, J. Neil Henderson, L. D. Carson, Norma Neely, Tewanna Edwards

**Affiliations:** 1 Department of Health Promotion Sciences, University of Oklahoma Health Sciences Center, Oklahoma City, OK, USA; 2 Department of Preventive Medicine and Public Health, University of Kansas, Kansas City, KS, USA; 3 Department of Biostatistics and Epidemiology, University of Oklahoma Health Sciences Center, Oklahoma City, OK, USA; 4 Tecumseh Early Head Start, Tecumseh, OK, USA; 5 American Cancer Society, Atlanta, GA, USA; 6 American Indian Institute, University of Oklahoma, Norman, OK, USA; 7 Oklahoma Health Care Authority, Oklahoma City, OK, USA

**Keywords:** breast cancer, mammography, participatory research, American Indians, Native Americans, theory of planned behavior, socio-ecological model

## Abstract

**Background:**

Breast cancer is an important public health issue among American Indian/Alaska Native (AI/AN) women in the US. This article describes the design and implementation of a culturally sensitive intervention to promote breast health among AI/AN women through a hybrid model that incorporates clinical and community-based approaches. This is one of the first studies using this model addressing breast cancer disparities among AI/AN populations in the US.

**Methods:**

The Theory of Planned Behavior was used as the guiding framework of the intervention and Community Based Participatory Research was the primary vehicle for the intervention planning and implementation. Three preliminary studies took place that aimed to identify qualitatively and quantitatively what deterred or encouraged AI women to get past or future mammograms. The research results were shared with community members who, through a prioritization process, identified the theoretical focus of the intervention and its corresponding activities. The priority population consisted of AI women ages 40–74, with no recent mammogram, and no breast cancer history.

**Results:**

The intervention centered on the promotion of social modeling and physician recommendation. The main corresponding activities included enhancing patient-physician communication about screening mammography through a structured dialogue, receipt of a breast cancer brochure, participation in an inter-generational discussion group, and a congratulatory bracelet upon receipt of a mammogram. Environmental and policy related changes also were developed.

**Conclusion:**

Creating a theory-based, culturally-sensitive intervention through tribal participatory research is a challenging approach towards eliminating breast cancer disparities among hard-to-reach populations.

## 1. Introduction

Breast cancer continues to be a major cause of death among American Indian/Alaska Native (AI/AN) women in the United States (US) [1]. Although the age-adjusted incidence rate among AI/AN women in 2012 was the fourth lowest at 96.9/100,000 among the five major ethnic groups in the US, mortality rates have declined among all racial/ethnic groups except for American Indians for the most recent 20-year time period [1]–[3]. Stage of diagnosis disparities also exist. Specifically, whereas 63% of Non-Hispanic Whites from 2003–2012 were diagnosed with localized cancer, only 58% among AI/AN women were diagnosed at a localized stage. Even more concerning is the disparity in metastatic breast cancer incidence. For women under the age of 50, only 4.5% of Non-Hispanic Whites were diagnosed with a metastatic stage compared to 6.8% of AI/AN breast cancer cases [2]. Another national study showed similar results. Specifically, AI/AN women had 2.3 fold higher odds of presenting with stage IV breast cancer compared with non-Hispanic White women, and 1.3 fold higher odds of being diagnosed with estrogen receptor (ER)-/progesterone receptor (PR)- breast cancer compared with non-Hispanic White women [4]. In other words, AI/AN women are more likely to be diagnosed with late stage breast cancer and with a more aggressive form of breast cancer. These disparities are due to a combination of factors related to socioeconomic status, access to health care, cultural differences and cancer biology. Although it is not clear which of the above factors contributes the most in the breast cancer disparities identified among AI/AN women, low mammography utilization could be a factor.

Nationwide, there is no consensus regarding the current mammography screening rates among AI/AN women. According to the American Cancer Society, AI/AN women 45 years and older have the lowest (61%) mammography screening rates within the past two years [1]. According to the Indian Health Service Government Performance and Results Act (GPRA) report of 2015, only 54.2% of all AI/AN women who were active clinical patients aged 52–64 years had a biennial mammography screening [5].

In Oklahoma, where the study takes place, the mammography screening rate among AI/AN women was 55% in 2010 [6]. The incidence rate of breast cancer among AI/AN women was 140.5/100,000 compared to 121.5/100,000 among Non-Hispanic White women for 2007–2009 [7]. Moreover, during the same period 34.2% of breast cancer diagnoses among AI/AN women were late stage, with regional or distant stages, compared to 31% among Non-Hispanic White women, however, this disparity was not statistically significant (*p* = 0.097) [7]. Based on a more recent study, AI/AN and white women in Oklahoma had similar 5 year observed survival rates (79.4 % and 80.2% respectively), however, within the 40–49 year old group, the survival rates were significantly lower among AI/ANs (*p* < 0.0001) [8]. Another recent study in Oklahoma found that among AI/AN women there is a geographical variation in breast cancer mortality, incidence and late stage diagnosis [9]. These epidemiological facts indicate that progress was made in reducing breast cancer among AI/AN women in Oklahoma; however, disparities still exist. Therefore, more intense efforts at the local level are needed in further reducing breast cancer disparities within the AI/AN female population by enhancing access to health care and increasing mammography screening.

There are very few interventions in the AI community promoting mammography screening that have been published. Studies have focused primarily on outreach activities [10]–[13], and the use of patient navigators or community health representatives [14],[15]. These abovementioned studies, however, only addressed two levels of the socioecological model [16]; the individual behavior and the interpersonal behavior or social networks. A socio-ecological approach to health promotion planning is essential due to the complexity of factors associated with mammography screening. Moreover, based on a recent meta-analysis paper, breast cancer screening programs targeting multiple leverage points are most effective for sustained outcomes [17].

We attempted to fill this research gap by developing a theory-based culturally-sensitive intervention based on the socioecological model [16] to promote breast cancer screening among AI women who live in a non-reservation setting. This study is one of the first to use a hybrid model in the development of an intervention that incorporates clinical and community-based approaches. In a recent report by the Institute of Medicine [18] the hybrid model is reported to be a promising approach toward the reduction of health disparities. In addition, the project was implemented through a community-based participatory research (CBPR) paradigm [19]. CBPR is an appropriate methodology to use when conducting research with AI communities since historically the lack of community involvement and inclusion has been viewed as exploitative, unethical and a possible cause of failed interventions [20]. Several studies with AI populations have successfully employed a CBPR approach [21],[22].

This article describes the planning process of a culturally-sensitive intervention based on the results of three preliminary studies. It also describes the products of the planning process including intervention activities. Moreover, this article highlights the importance of using a socio-ecological framework and CBPR approaches in the development of the intervention.

## 2. Methods

*Intervention setting*: The study took place at a single tribal clinic and its broader tribal jurisdictional area which encompasses an area of 900 square miles. The tribal clinic serves all AI/AN women who live in the jurisdictional area. In 2007, during the early phases of the project, the tribal clinic served 12,000 patients with a total of 74,000 visits. Eight hundred forty-nine breast cancer referrals were written and among those women who were referred, 611 (72%) women had a screening mammogram. In fact, the mammogram screening rates for female patients ages 52 to 64 have been declining; 81.1% in 2005, 74.5% in 2006, 72% in 2007 and 35.3% in 2008. The tribal leadership requested that the authors not reveal the name of the tribe. This tribe, like all American Indian nations, is comprised of a cultural mix of membership. The cultural mix ranges from those who are quite proud of their tribal membership but do not speak the language or have deep inherited enculturation experience, to those who do have such experience. In most ways, tribal members are participants in general American culture and to varying smaller degrees, in their tribal culture. The priority population consisted of AI/AN women residing in this tribal jurisdictional area, 40–74 years old, who did not have a screening mammogram within the last two years, and who were never diagnosed with breast cancer.

At the time of planning the intervention in 2010, and based on the clinic's medical records, 547 (55%) eligible women for mammography screening did not have a mammogram within the last two years. During the early stages of the study, the medical leadership of the clinic followed the mammography screening guidelines recommended by the American Cancer Society, which recommended an annual screening mammogram for all women 40 years of age and older [23]. However, in December 2011, there was a change in the clinic's medical leadership administration. The new medical director followed the U.S. Preventive Services Task Force (USPTF) guidelines, which indicate a biennial screening mammogram starting at the age of 50 [24] and therefore he encouraged all the medical providers at the clinic to also follow these new guidelines.

**Figure 1.**
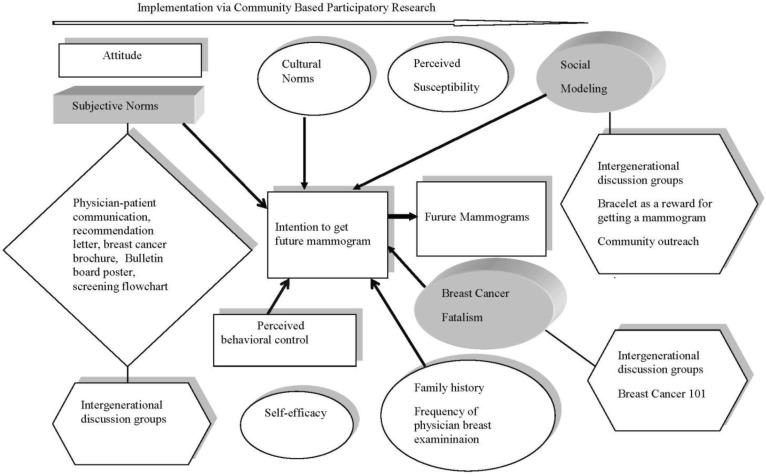
Theoretical framework: The proposed expanded model of the Theory of Planned Behavior (TPB) and the hybrid intervention approach. The squares refer to the TPB, the circles to the secondary constructs, the 3-D diagrams to the constructs addressed in the intervention, the diamonds to the clinic-based activities and the hexagons to the community-based activities.

*Theoretical framework*: In this study, we used an integrative conceptual framework that incorporated elements from the Theory of Planned Behavior (TPB) [25],[26], Social Cognitive Theory (self-efficacy and social modeling) [27], the Health Belief Model (perceived susceptibility) [28] as well as concepts that have been shown consistently to be related to mammography screening such as fatalism [29]–[31] and cultural norms [32]. The TPB [25],[26] was used as the primary conceptual model for the development of the assessment survey developed in the early phase of this project, called the Women's Health Survey (WHS) [29]. The theory posits that intention is the immediate antecedent of behavior and it is assumed to capture the motivation to behave in a particular way. According to the TPB, intention is determined by three factors: attitude toward the behavior, subjective norms (i.e. social norms), and perceived behavioral control (i.e. perceived ease or difficulty of performing a behavior). A depiction of the theoretical framework can be found in Figure 1.

*Community Based Participatory Research*: CBPR was implemented with the use of a community steering committee (CSC) and of community research assistants (CRAs). The CSC consisted of lay people in the community, breast cancer survivors, tribal clinic representatives, representatives of other local coalitions and other state organizations. Under the guidance of two CBPR experts during the first two years of the project, the CSC underwent two training workshops which consisted of the following: (1) a review of basic community social and political segments common to all communities; (2) an overview of the CBPR concept; (3) the techniques of implementing, conducting, and maintaining CBPR initiatives; and (4) the basic methods of CBPR processes and outcomes evaluation. The CSC used consensus as the method for reaching group decisions. Adhering to the principles of CBPR, the CSC was committed to being integrally involved in all phases of the study, which included data collection, analysis and interpretation. The CSC met on a monthly basis to discuss strategies required to carry out the program activities. Other ways that we promoted active participation and trustworthiness, were by: (a) keeping minutes of the meetings and following through with the action items at the end of the meeting; (b) seeking input on important decisions from all stakeholders involved even among those who were are not present at the meeting; (c) having the project coordinator develop the agenda and lead the steering committee meetings; and d) using small group work [33].

Another mechanism that we used to ensure that research was carried out by community members was the use of CRAs. CRAs are a group of paid community researchers who were trained in research methods including human research subjects projection, data collection, data entry, and data analysis. In addition to the initial training in CBPR, they also attended trainings in qualitative research methods that focused on how to interview study participants, conduct focus groups, and analyze qualitative data.

*Formative research*: Three preliminary studies led to the development of this intervention. During the first study (2005–2006) we conducted key informant interviews, elicitation interviews, and focus groups with AI women to identify the motivational beliefs affecting their decision to obtain or not obtain a mammogram. Most women expressed mixed attitudes toward mammography with prominent beliefs about fear and pain associated with mammography. Participants also noted the inclusion of family, friends and personal physicians as critical social referents. Moreover, they indicated environmentally related factors such as scheduling procedures as barriers toward regular mammography screening. Culturally related beliefs identified were centered on the use of traditional medicine and the role of AI woman in the broader AI community [34].

The results obtained through the qualitative research were used to develop the WHS that would measure the prevalence and relative importance of AI women's motivation to undergo a screening mammogram. The second preliminary study consisted of the administration of the WHS prospectively through the use of a stratified random sample (n = 255) [29]. Results indicated that physician recommendation, being knowledgeable about mammography screening, and receiving an annual Professional Breast Exam (PBE) were predictors of past mammography experience, whereas fatalistic breast cancer attitudes and higher perceived barriers toward getting a mammogram were deterrents of past mammography experience [29]. Additional data analysis revealed that intention to get a future mammogram in the next 6 months was associated with social modeling (odds ratio OR = 1.57, 95% CI: 1.13–2.18) (i.e. women who made known to other women that they got a mammogram or have been an example to other women regarding mammography), and family encouragement to get mammograms (OR = 1.06, 95% CI: 1.00–1.12). Moreover, breast cancer fatalism was marginally negatively associated with age (*p* = 0.08), but significantly negatively associated with knowledge about mammography screening (*p* = 0.0008) [29].

The results of the quantitative research were shared with the CSC. Jointly, the research team and the CSC decided to conduct more qualitative research to determine how the broader community felt about the results and if there were additional issues to consider in the final stage of intervention planning. This led to the third preliminary study (2012–2013).

We conducted three focus groups (n = 14) with AI women and 16 key informant interviews. Focus group participants were identified via a computerized list of eligible participants for mammography, kept at the tribal clinic. A random sample was selected to obtain a variety of opinions. Inclusion/exclusion criteria were that participants: (a) do not have a history of breast cancer; (b) did not participate in the preliminary studies that were conducted in 2005–2006 or 2011–2012; and (c) aged 52–74. Qualitative data analysis was performed using standard content analysis techniques [35],[36].

The results of the third study corroborated those of the other two preliminary studies. AI women and key informants stressed the importance of a more productive communication between the physician and patient regarding the topic of breast cancer and screening mammography. Additional suggestions included better reminder and follow-up systems in relation to mammography appointments, initiating a dialogue between older women and younger women about the importance of mammography and sharing prior experiences of mammography screening among AI women.

The results of the third study were communicated back to the CSC along with the results of the other two preliminary studies to determine the foci of the intervention. Information about effective strategies that took place among other AI communities for breast health outreach [14],[15] also was communicated to the CSC. The community members proposed a list of activities that corresponded to each selected construct. Each CSC member also was asked to rate each proposed activity based on the principles of importance and feasibility [37]. All the ratings were summarized by developing a 2×2 table with four quadrants. According to the Precede/Proceed Model [37], activities that fall into the most important/most feasible category/quadrant are the ones selected for interventions.

During the planning stage and throughout the study, the academic researchers paid also attention to their community entrée and gaining trust from the community [38]. Transparency, open communication, face-to-face meetings, and participation in community events were some of the tools that the research team used to nurture this trust. Several community partners were involved and provided resources such as staff and space for meetings. To further promote shared identity and cohesiveness among all partners, collaboratively we created a mission statement, a project logo, a motto statement, and a T-shirt that listed the names of all partners. The name of the project is “Native Women's Health Project”, and our mission statement is “To save American Indian women's lives from breast cancer by promoting routine breast cancer screening through education and policy changes with a commitment to cultural sensitivity, respect and compassion”. Our motto is “Shared Love from Generation to Generation”. Our logo depicts the medicine wheel, which is a powerful symbol within the AI culture symbolizing harmony and connections among all living beings on Earth with a pink ribbon in the middle and two eagle feathers hanging from the bottom of the medicine wheel.

All study participants, throughout the three preliminary studies completed an informed consent form, which was approved by the University of Oklahoma Institutional Review Board, and were offered a $20 gift card as a reimbursement for their participation in the project.

## 3. Results

Upon reviewing the results of the qualitative and quantitative research, as well as through additional brainstorming, the research team and the CSC developed 19 programmatic activities corresponding to eight different concepts (Table 1). Based on the prioritization process [37] the activities that were selected as most important and most feasible were: (a) improve communication between patient/physician about mammography screening; (b) make information about breast health more accessible to AI women in locations they are more likely to congregate; (c) provide counseling regarding mammography results regardless of outcomes; and (d) encourage breast self-examination by using breast models. These initial strategies were further discussed. We decided, for example, that the program should not promote breast self-examination since this was no longer a valid recommendation by the ACS [39] or the USPSTF [24]. Instead, we decided to focus on educating women about being aware of changes in their bodies especially in relation to the signs and symptoms of breast cancer. We also decided not to focus on promoting counseling on mammography results because additional counseling resources were not available at the clinic. We also agreed that no emphasis would be put on promoting PBE by the medical providers despite the fact PBE seemed to be an important motivating factor according to the quantitative research results. Instead, the CSC decided that it was up to the patient to request a PBE. Other important concepts which were highlighted via the quantitative research such as social modeling and breast cancer fatalism were also revisited during our discussions.

**Table 1.** Proposed activities based on research findings and discussions with steering committee members.

Concept	Proposed Activity	
*Social Modeling*	1.	Community Health Representatives/One-on-One approach with women at their homes who never had mammograms
	2.	Talking Circles on mammography/breast health with women of all ages
	3.	Promote awareness on breast health through arts and crafts
	4.	Promote awareness on mammography via bulletin boards
	5.	Use of Mobile Mammography Units
	6.	Use of word of mouth of breast cancer survivors to promote breast health awareness
	7.	Encourage women to develop a buddy system (go and get a mammogram together)
*Physician influence*	8.	Improve communication between patient-physician on mammography screening, more information on what is mammography, why it is important, develop a checklist for physicians to use / talking points
	9.	Reach consensus on screening recommendations
*Barriers to mammography screening/anxiety related to waiting for mammography results*	10.	Shorten the time between getting a mammogram and getting the results of the mammogram
	11.	Provide counseling regarding the mammography results regardless of the outcome
*Facilitators-reminder system, someone setting up the mammogram*	12.	Improve existing reminder system to have a repeat mammogram such as a check list for physicians and nurses on the patient's chart
*Social support*	13.	Develop a website for breast cancer survivors and exchange of information about breast health
*Social influence from family and friends/encourage younger women to get mammograms*	14.	Through Talking Circles, encourage breast self- examination-purchase breast models and show women how to use them
	15.	Make a bead bag or a leather pouch that resembles a breast with lump as a reminder to do mammograms
*Role of culture, past experiences with mistreatment at health care systems, remembrance of standing in lines for commodities*	16.	Provide a culturally tailored video about mammography for women while they are waiting for their doctor's appointment
*Enhance knowledge on breast health and mammography screening*	17.	Make the information on breast health more accessible and possibly develop a display in locations where women are likely to congregate
	18.	Link with existing local organizations and have information booths during sponsored events (pow wows, stomp dances, festivals)
	19.	Develop a Facebook account/brochure/logo for the project, to increase awareness about the project

At the end of this iterative planning process, the CSC ultimately decided that the program would focus on the following areas: (a) promoting social modeling to overcome fear; (b) improvement of patient-physician communication; (c) clarification of breast screening recommendations and enhancement of knowledge about mammography screening guidelines; (d) enhancement of social support; (e) promotion of social influence from family and friends, particularly encouraging daughters or younger women to get mammograms; and (f) decreasing breast cancer fatalism. A Logic Model was developed that served as a blueprint of the implementation of the intervention.

As stated earlier, one of the unique features of this project was the incorporation of a hybrid model for intervention. The clinical component was centered on the role of the physicians/medical providers as gatekeepers of mammography screening. Through separate meetings with the medical director and clinic staff, we attempted to identify first, what was currently done in regards to physician recommendation, and second, what could be changed to improve current practices. We also took a comprehensive approach by emphasizing all aspects of the current clinic practices (i.e. physician recommendation, clinic policies, reminder and effective communication systems) [40]. For instance, to strengthen the patient/physician communication the physicians indicated the lack of patient aides that are readable and attractive to the patient. As a response to this need, we developed a brochure on breast cancer and mammography screening that was given to the patient upon completion of her visit. In addition to providing medical facts about breast cancer and mammography, the brochure included a testimonial by a breast cancer survivor along with a statement of how early detection of breast cancer saved her life. The use of a testimonial is a form of story-telling and it can serve as a catalyst for changing health behavior by arousing not only a cognitive but also an emotional response from the reader [41],[42].

Another issue that arose during our conversations with the physicians was the insufficient time during the visit with the patient to discuss screening mammography. As a response to this need and based on the Precaution Adoption Process Model [43] we developed a brief questionnaire that helped the medical provider identify the patient's stage of the decision-making process, and thus initiate a conversation on mammography screening tailored to her needs.

Another need identified was the lack of a standardized approach to mammography screening recommendation. Therefore, we developed an algorithm or a flowchart for screening mammography that was posted in the exam rooms. The flowchart was designed to assist medical providers in their decision making process of recommending mammography to patients by assessing the patient's family risk in addition to other criteria such as genetic predisposition, personal history of breast cancer and having dense breast tissue.

Finally, in order to further strengthen the current reminder system we developed a bulletin board poster encouraging women about mammography. The poster stated that an AI woman should get a mammogram for her own peace of mind and to be able to live longer and see her grandchildren grow. These health communication messages were attitudinal beliefs expressed during the qualitative research which were further transformed into belief statements on the WHS. These two attitudinal beliefs, “help me leave longer and watch my children and grant-children grow” and “give me a peace of mind to find out that I am healthy” had the highest correlation with past mammography experience (*p* = 0.0038 and *p* = 0.0012 respectively), and thus chosen to be transformed into health communication messages.

**Figure 2.**
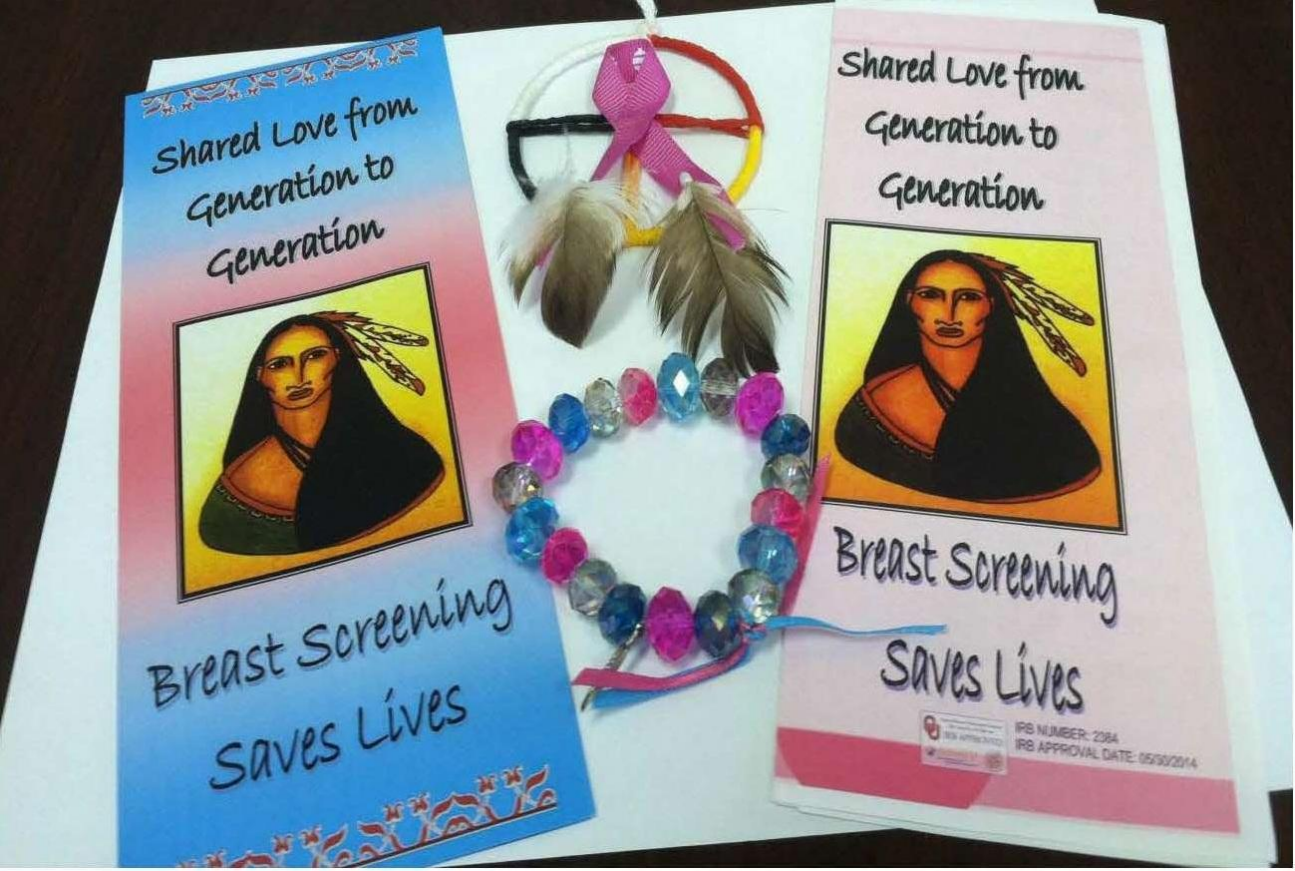
Culturally sensitive brochures on mammography screening and the bracelet given upon receipt of a mammogram that were developed for the Native Women's Health Project. You can also see the logo of the project.

The second part of the hybrid approach is the community component which was centered primarily on the construct of social modeling. Several intervention strategies took place to promote social modeling. The first one was the development of bracelets by a local AI artist. These hand-made of three colors (pink, white and turquoise) bead bracelets were given as rewards to the women for getting a screening mammogram. It was hypothesized that the bracelet will act as a catalyst of discussion among women on breast cancer/mammography and that other women will be encouraged indirectly to get a mammogram. You can see a picture of the bracelet and other educational material in Figure 2.

The second intervention strategy was the creation of intergenerational group discussions during which AI women from our priority population along with a younger person (e.g. daughter and niece) were invited to participate. The discussion group incorporated elements of the Freire approach of raising consciousness [44]–[46], storytelling [47] and an intergenerational approach to health [48],[49] methodologies.

A 10-minute video-clip from a video called *The Shawl Project* was shown. The “Shawl Project” is a project among AI breast cancer survivors who created pink shawls (shawls are of great significance in the AI culture and signify protection and care of children by their mothers) not only for themselves, but also for their daughters. The messages that the video conveyed were the importance of regular screening, knowing one's body and passing these preventive breast health messages as an act of care from the older generation of AI women to the younger generation, the same way a mother would give to her daughter a pink shawl. The discussion took place in a non-threatening and a non-judgmental environment. In line with the Freire methodology the video clip acted as a “code” that triggered a structured discussion around the issue of breast cancer. The discussion started by first reflecting on the broader AI community's status and relationship with breast cancer, then moved to a more personal level and ended with a menu of action steps suggested by the women themselves. We conducted six discussion groups. Twenty-six participants and guests participated in the discussion groups. Highlights of the intergenerational discussion groups are shown in table 2.

**Table 2.** Representative participants' comments from the inter-generational discussion groups.

Question	Representative comments
*What do you see in the “Pink Shawl” video*?	“Group support and protection”
	“Symbolism of Pink Shawl-support”
	“Unity”, “Embracing all people”
	“Women empowering each other”
*What is really happening in the video?*	“People don't want to talk about it (breast cancer) even if they have breast cancer”
	“Women are passing knowledge from generation to generation trying to prevent it (cancer)”
	“Awareness. Cancer awareness is being brought to everyone; not just the sick”
*How do these stories of women who are breast cancer survivors relate to our lives*?	“My sister had breast cancer and didn't tell anyone about it for a long time. I now check on her all the time”
	“Helps me to start talking with other women about breast cancer”
	“Mother didn't go (to the doctor), didn't want to know (if she had breast cancer), wanted to tell.”
	“Encouragement. Being open about it. Getting together helps to talk about it.”
*Why does breast cancer exist among Native American women?*	“Not go to the doctor”
	“I don't want to burden my husband”
	“Fear to find out if they (American Indian women) have cancer”
	“Automatic death sentence, no hope, not know they (American Indian) don't have to die from it”
	“Native American women not assertive enough to ask questions or be in charge of personal health ”
	“Smoke Shops. We smoke and we know we shouldn't smoke, but we ignore it and do it anyway.”
	“Native women are more reserved, hesitant to discuss such issues”
	“If you know, you go ( i.e. die) faster”
	“Higher risk”
*How can Native American women become empowered now that we better understand the breast cancer problem?*	“Start talking about it”
	“Do more group discussions”
	“Start talking to our daughters and granddaughters”
	“Why can't we help the husbands understand how important this is?”
	“Spread the word. Talk to everyone.”
*What can we do to reduce breast cancer among Native American women?*	“Have discussion groups with elderly- learn and share information; small groups”
	“Healthy food”
	“Personalized one-on-one approach”
	“Early detection”
	“Support groups”
	“I need someone to go with me (for a mammogram)”
	“Faster results/make it easier to get a mammogram”
	“Go out to schools-talk about it (breast cancer) to young women”
	“Talk about it (breast cancer) more in the clinic/family”

The inter-generational discussion groups were also helpful in addressing the construct of breast cancer fatalism. By targeting not only women who are eligible for mammography, but also younger women who might also share these fatalistic attitudes we hoped to raise consciousness about breast cancer and the importance of mammography among the younger generation. In addition, as guided by the formative research and particularly the strong negative association between breast cancer fatalism and knowledge of mammography screening, we also educated the participants, through interactive activities, about the myths of breast cancer and its signs and symptoms.

**Figure 3.**
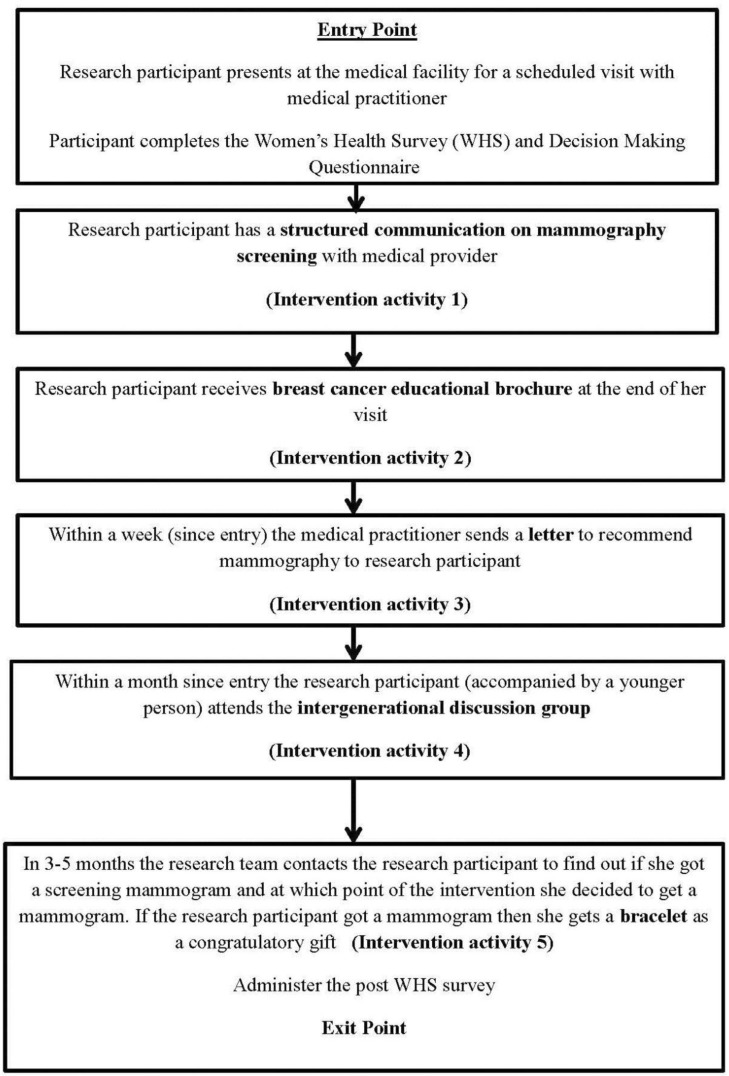
Flow of the intervention activities of the Native Women's Health Project.

A main consideration in the design of the intervention was that each intervention activity was integrated in the context of the AI women's lived experiences. It was imperative that all pieces were logically connected with each other, so that the intervention would run smoothly. At the entry point of the intervention the participant engaged in a structured communication with the medical provider. This was followed by a discussion of the breast cancer brochure. Within a week, the medical practitioner would send a recommendation letter to the patient. Within a month, the participant would be invited to one of the two scheduled intergenerational discussion groups. Finally, upon receiving a mammogram a congratulatory gift (i.e. the bracelet) was given to the participant. A depiction of the overall intervention can be found in Figure 3. Additional information about the educational material and products developed via this intervention can be given from the first author.

## 4. Discussion

In this study we were able to build a culturally-supported intervention from the ground-up by combining research results with local knowledge and expertise. In the fight to reduce cancer disparities among AI/ANs, researchers need to work at both the macro and the micro level [50]. At the macro level we refer to efforts taken at the federal level such as increasing funding on mammography screening through the Indian Health Services and the National Breast and Cervical Cancer Early Detection Program [51]. At the micro level we refer to efforts taken at the local level promoting education and policy/environmental changes conducive to mammography screening. This intervention is a step towards decreasing health disparities at the micro level.

This study is unique because it is one of the few comprehensive studies, to the best of our knowledge, that utilizes the socio-ecological approach to the planning of a health promotion intervention among AI/AN women with a focus on early detection and prevention of breast cancer. As noted earlier, due to the complexity of barriers related to mammography screening, it was imperative the project utilized a socio-ecological approach with equal emphasis placed on the individual, community systems and environmental factors that might contribute to reduced mammography rates in the community. In our study, the individual level was addressed through the delivery of a breast cancer brochure to the patient and through face-to-face structured communication between the medical provider and the patient. The interpersonal level was addressed by giving a bracelet as a congratulatory gift for getting a mammogram and through the inter-generational discussion groups where the “Shawl Project” video was the stimulus for discussion. The policy level was addressed through the development of clinic policies and particularly through the use of the mammography screening algorithm. The community level was addressed by building partnerships among academia, community-based organizations, tribal authorities and clinic services. Environmental changes also took place through the mammography screening poster on one of the clinic's bulletin boards. Using the socio-ecological model as a blueprint for the design of this intervention is a complex process but at the same time a potentially promising approach toward decreasing mammography disparities. Evidence stemming from previous work in tobacco control shows that comprehensive programs that incorporated clinical and community strategies were effective in decreasing tobacco use [52].

In fact, this study can potentially contribute to the literature of evidence-based interventions promoting mammography screening among AI/AN populations. Currently, according to the Community Preventive Services Task Force (i.e. Task Force) (http://www.thecommunityguide.org/cancer/index.html) effective interventions promoting breast cancer screening based on a systematic literature review process include the following: client reminders, one-on-one education (e.g. lay health advisors), group education, reducing out-of-pocket costs, reducing structural barriers (e.g. use of mobile mammography clinics) and medical provider assessment and feedback [53]. However, very few of the studies reviewed by the Task Force targeted hard-to-reach (e.g. women who never had mammograms) and/or AI/AN populations. In the absence of such research, this study attempted to fill this research gap by implementing both client and provider based approaches, with a broader emphasis though on the community and the clinic's health care system, among AI/AN women who were overdue for mammography screening. Future impact evaluations of this intervention will demonstrate if indeed it is effective in promoting breast cancer screening among AI/AN women.

The use of CBPR was instrumental in the development of this intervention. CBPR was implemented by employing AI community residents as research staff, utilizing a team approach in research decision making and practice, a joint program-research oversight of the research process, sharing preliminary findings with community partners, and engaging them in interpretation of findings and implications for program practice [54]. CBPR also was implemented by adapting the project to the cultural norms and policies of the community it served. For instance, during the last phase of the project (2011–2015) the tribal clinic adopted the USPSTF screening guidelines and abandoned the ACS related guidelines. This caused a change in the study's research design and particularly in the upper and lower age limits of the women recruited in the study, since the initial intention was to use the ACS guidelines. The research team decided to relax the rigor of the research design in order to respect the norms and policies of its main collaborative partner.

Another example refers to the development of the discussion groups. Although the literature states that Talking Circles [55],[13] are a culturally appropriate vehicle for discussions among AI/ANs, we decided not to do that as the community partners felt this format was too rigid and not in line with the cultural norms of this group. A third example refers to the use of clinic-based community health representatives as a proposed strategy to educate AI women on mammography screening in their own homes and encourage them to get mammograms. Unfortunately, due to other priorities at the time, the clinic could not support this strategy, despite the fact that patient navigation on breast screening is considered an effective practice in increasing mammography screening among AI/AN populations [14],[15]. Therefore, the research team abandoned this strategy and instead, we used group oriented health education approaches. Listening to the community in every step of project implementation was instrumental in ensuring that the intervention would be accepted by all community partners. Our study provides support to the existing literature that states that successful community-based research within the AI/AN populations cannot be implemented without the use of tribal participatory research [54],[56].

A similar concept to CBPR was the utilization of a strengths-based approach [56]. By doing so, the local community is more likely to invest themselves and their resources in this project and the products of this collaborative are more likely to be sustainable [57]. This was evident during our work with the tribal clinic representatives. By doing formative research we built on existing practices or capacities of the tribal clinic and we attempted to understand the local context where the intervention would take place before we developed any intervention strategies. Working with tribal clinics can be challenging due to competing priorities and time constraints. Therefore, it was important that we created intervention activities that were feasible and useful to the clinic and its patients. The ongoing use of the screening algorithm or flowchart and the use of the bulletin board at the clinic despite the fact that the project has officially ended, is an outcome of this early collaborative approach.

With the current emphasis on translating research findings into to practice and policy statements, there is a gap as to how this is achieved especially in community-based settings. The iterative process that we used in this study by combining research results, with community wisdom and past effective practices was central in developing products that were unique to this community. The use of qualitative research throughout the planning of the initiative was also instrumental in giving the community several opportunities to reflect on the research findings and voice their concerns and suggestions. Another reason for taking into consideration the community's wisdom is that AI communities are very diverse and unique, and what is salient to one community may not be salient to another [58].

From a theoretical perspective this study is unique because it is one of the first that applied the expanded behavioral theoretical framework of the TPB [25],[26] among AI women. There are very few interventions in the AI community promoting mammography screening based on a sound theoretical/behavioral framework. The most common theoretical constructs used were self-efficacy [14] and social support [12]. Interventions using a theoretical framework are most effective in increasing breast cancer screening rates [59]. In addition, the theoretical framework was used in this study, not only to identify targets for behavioral changes or to predict future mammography behavior, but more importantly to guide the development of the intervention implementation and its subsequent evaluation. Very few studies have used this approach [60],[61].

Finally, the application of the Freire methodology of raising consciousness was another unique feature of this project that helped women to move from a stage of passivity to a stage of action through a group discussion. The first author had the opportunity to attend all discussion groups and she can attest that the process was indeed transformative as most women by the end of discussion indicated that they would have a mammogram. Very few studies [62],[63] have used the Freire methodology of critical consciousness among AI populations. We strongly recommend that more researchers working with AI/ANs utilize this methodology not only in the area of breast health but with other health topics as well.

## 5. Conclusion

The collaborative nature of CBPR provided a more comprehensive and accurate framework for understanding AI/AN women's and the broader community's beliefs about mammography screening which led to the development of a culturally-based intervention including policies and environmental changes that still benefit the community. Traditional methods of research could not have produced outcomes that were as translatable or as meaningful to the community. By incorporating the community's input we promoted ownership of the intervention by the community members, which further promoted the long-term sustainability of the project even when the research ended. The use of a theoretical framework was instrumental in the design of the intervention as well as during its implementation. Theoretical frameworks accompanied by tribal participatory research can enhance the quality of program implementation. Utilizing the socio-ecological approach and implementing a hybrid model where emphasis is put on both community- and clinic-based interventions is a challenging and complex process. However, it is the only approach in developing long-term and sustainable interventions.
